# The Regulation of Cbf1 by PAS Kinase Is a Pivotal Control Point for Lipogenesis *vs.* Respiration in *Saccharomyces cerevisiae*

**DOI:** 10.1534/g3.118.200663

**Published:** 2018-10-31

**Authors:** Desiree DeMille, Jenny A. Pape, Benjamin T. Bikman, Majid Ghassemian, Julianne H. Grose

**Affiliations:** * Department of Microbiology and Molecular Biology; † Department of Physiology and Developmental Biology, Brigham Young University, Provo, UT 84602; ‡ Department of Chemistry/Biochemistry, University of California at San Diego, La Jolla, 92093-0378

**Keywords:** PAS kinase, Cbf1, USF1, respiration, lipogenesis, mitochondria

## Abstract

PAS kinase 1 (Psk1) is a key regulator of respiration in *Saccharomyces cerevisiae*. Herein the molecular mechanisms of this regulation are explored through the characterization of its substrate, Centromere binding factor 1 (Cbf1). *CBF1*-deficient yeast displayed a significant decrease in cellular respiration, while PAS kinase-deficient yeast, or yeast harboring a Cbf1 phosphosite mutant (T211A) displayed a significant increase. Transmission electron micrographs showed an increased number of mitochondria in PAS kinase-deficient yeast consistent with the increase in respiration. Although the *CBF1*-deficient yeast did not appear to have an altered number of mitochondria, a mitochondrial proteomics study revealed significant differences in the mitochondrial composition of *CBF1*-deficient yeast including altered Atp3 levels, a subunit of the mitochondrial F_1_-ATP synthase complex. Both beta-galactosidase reporter assays and western blot analysis confirmed direct transcriptional control of *ATP3* by Cbf1. In addition, we confirmed the regulation of yeast lipid genes *LAC1* and *LAG1* by Cbf1. The human homolog of Cbf1, Upstream transcription factor 1 (USF1), is also known to be involved in lipid biogenesis. Herein, we provide the first evidence for a role of USF1 in respiration since it appeared to complement Cbf1 *in vivo* as determined by respiration phenotypes. In addition, we confirmed USF1 as a substrate of human PAS kinase (hPASK) *in vitro*. Combined, our data supports a model in which Cbf1/USF1 functions to partition glucose toward respiration and away from lipid biogenesis, while PAS kinase inhibits respiration in part through the inhibition of Cbf1/USF1.

Proper resource allocation is fundamental to the success of any system. In cellular organisms, it is crucial to sense available nutrients and astutely allocate them among several pathways including growth, storage and energy metabolism. If nutrients are not properly allocated, *e.g.*, when too many nutrients are diverted to one pathway, it comes at the expense of another important pathway and often leads to diseases such as obesity, diabetes and cancer. One of the mechanisms that cells have evolved to help coordinate resource allocation are nutrient sensing protein kinases (Lindsley and Rutter 2004).

PAS kinase is a highly conserved sensory kinase with both a sensory PAS (Per-ARNT-Sim) domain and a serine/threonine kinase domain (Rutter *et al.*, 2001). It is a key player in sensing and allocating glucose in eukaryotic cells (reviewed in Cardon and Rutter 2012; DeMille and Grose 2013; Grose and Rutter 2010; Hao and Rutter 2008; Schlafli *et al.*, 2009; Smith and Rutter 2007; Zhang *et al.*, 2015). Additionally, PAS kinase is activated both in yeast and mammalian cells under conditions that activate respiratory metabolism (da Silva Xavier *et al.*, 2004; Grose *et al.*, 2007). This occurs in yeast when cells are grown on carbon sources other than glucose and in mammalian cells under conditions of high glucose.

PAS kinase is not only activated by respiratory conditions, but is also implicated in regulating respiratory metabolism itself. PAS kinase-deficient mice (PASK^−/−^) have a hypermetabolic phenotype in that they consume more oxygen and give off more CO_2_ and heat when placed on a high-fat diet (Hao *et al.*, 2007). In contrast, PASK^−/−^ mice also accumulate significantly less hepatic lipids when placed on the high-fat diet and PAS kinase knockdown or inhibition also decreases triglyceride in hepatic cell lines (Wu *et al.*, 2014). These data suggest that PAS kinase is allocating glucose away from respiration toward lipid storage in wild type mice. However, the mechanisms behind these phenotypes are largely unknown. We recently identified a novel substrate of yeast PAS kinase 1 (Psk1), Centromere binding factor 1 (Cbf1), that could be an important player in the mechanisms behind these phenotypes (DeMille *et al.*, 2014).

Cbf1 is a general transcription factor conserved from yeast to man (Baker *et al.*, 1989; Bram and Kornberg 1987; Cai and Davis 1990). Its human homolog, Upstream transcription factor 1 (USF1), is a key regulator of genes involved in lipid homeostasis and USF1 mutations are strongly correlated with hyperlipidemia (Auer *et al.*, 2012; Di Taranto *et al.*, 2015; Laurila *et al.*, 2016; Naukkarinen *et al.*, 2006; Pajukanta *et al.*, 2004). In yeast, transcriptome data suggests that Cbf1 regulates a wide variety of genes including those involved in respiration and lipid biogenesis, as well as amino acid biosynthesis (Cai and Davis 1990; Kolaczkowski *et al.*, 2004; Lin *et al.*, 2013). We recently provided evidence for decreased respiration in *CBF1*-deficient yeast and for the PAS kinase-dependent phosphorylation and inhibition of Cbf1(DeMille *et al.*, 2014). The altered respiration observed in yeast lacking *CBF1* or PAS kinase may be due to many effects within the cell such as 1) effects on total mitochondrial mass or 2) electron transport chain expression or activity. Here we further characterized the mechanisms behind PAS kinase and Cbf1 respiratory function in yeast. Specifically, we report the differences observed in mitochondrial area between wild type, *CBF1*-deficient and PAS kinase-deficient yeast and present the mitochondrial proteomes of these yeast. In addition, we confirm the role of *Cbf1* in regulating the yeast lipid genes *LAC1* and *LAG1* and provide evidence that they are downregulated by *Cbf1* under the same conditions that *ATP3* is upregulated. Evidence is also provided for USF1 being a conserved PAS kinase substrate through *in vitro* kinase assays as well as yeast complementation assays. Combined, our data supports a model in which Cbf1/USF1 partitions glucose toward respiration at the expense of lipid biogenesis, while PAS kinase inhibits Cbf1/USF1 favoring lipid biogenesis.

## Materials and Methods

### Growth assays and vector construction

A list of strains, plasmids and primers used in this study are provided in Table 1. All plasmids constructed for this study were made using standard polymerase chain reactions (PCR) followed by restriction digests using enzymes from New England Biolabs (Mymrikov *et al.*, 2011). Human USF1 was PCR amplified using primers 3456/3457 from plasmid pCMV6-USF1 (OriGene, SC 1227700). *CBF1*-deficient yeast acquire suppressors at a rapid rate. Therefore, all cultures were streaked fresh from frozen and used as quickly as small colonies appeared.

**Table 1:** Strains, plasmids and primers used in this study

Strain	Background			Genotype		Abbreviation		a/α	Reference or source
JGY1	W303			*his3*, *leu2*, *lys2*, *met15*, *trp1*, *ura3*		WT		a	David Stillman, University of Utah
JGY4	W303			*psk1*::his3, *psk2*::kan-MX4, *leu2*, *lys2*, *met15*, *trp1*, *ura3*		*psk1psk2*		a	(Grose *et al.*, 2007)
JGY43	BY4741			*his3-1*, *leu2-0*, *met15-0*, *ura3-0*		WT		a	(Winzeler *et al.*, 1999)
JGY1227	BY4741			*cbf1*::kan-MX4, *his3-1*, *leu2-0*, *met15-0*, *ura3-0*		*cbf1*		a	(Winzeler *et al.*, 1999)
JGY1244	BY4741			*psk1*::hph-MX4, *psk2*::nat-MX4, *his3-1*, *leu2-0*, *met15-0*, *ura3-0*		*psk1psk2*		a	This study
JGY1261	BY4741			*psk1*::hph-MX4, *psk2*::nat-MX4, *cbf1*::kan-MX4, *his3-1*, *leu2-0*, *met15-0*, *ura3-0*		*psk1psk2cbf1*		a	This study
JGY1348	BY4741			*psk1*::kan-MX4, *his3-1*, *leu2-0*, *met15-0*, *ura3-0*		*psk1*		a	(Winzeler *et al.*, 1999)
JGY1349	BY4741			*psk2*::kan-MX4, *his3-1*, *leu2-0*, *met15-0*, *ura3-0*		*psk2*		a	(Winzeler *et al.*, 1999)
JHG504	BL21DE3			F^-^ ompT hsdSB(rB^-^mB^-^) gal dcm (DE3)		BL21			Novagen

Plasmid	Gene		Description		Backbone	Yeast Origin	Selection		Reference or source
pJG121	EV		Empty pRS415 plasmid		pRS415	CEN	LEU		(Simons *et al.*, 1987)
pJG173	*PSK2*		pGAL1-10-PSK2-HIS/HA		pRS426	2u	URA		(Grose *et al.*, 2007)
pJG210	*UGP1*		Ugp1-HIS		pHIS-parallel		AMP		(Smith and Rutter 2007)
pJG725	EV		pAdh-myc		pRS416	CEN	URA		(DeMille *et al.*, 2014)
pJG858	PSK1		pGAL1-10-PSK1-HIS/HA		pRS426	2u	URA		(DeMille *et al.*, 2014)
pJG1009	EV		pET15b with James Y2H MCS		pET15b		AMP		(DeMille *et al.*, 2014)
pJG1025	*IPP1*		*IPP1* into pET15b (pJG1009)		pET15b		AMP		(DeMille *et al.*, 2014)
pJG1031	*CBF1*		*CBF1* into pET15b		pET15b		AMP		(DeMille *et al.*, 2014)
pJG1232	*USF1*		USF1 in pCMV		pCMV6-XL5		AMP		OriGene
pJG1233	*USF1*		USF1 into pET15b		pET15b		AMP		This study
pJG1246	*USF1*		USF1 into pJG725		pRS415	CEN	URA		This study
pJG1314	*LACZ*		*LACZ* into pJG121		pRS415	CEN	LEU		This study
pJG1315	*ATP3*		*ATP3-LACZ*		pRS415	CEN	LEU		This study
pJG1316	*COX4*		*COX4-LACZ*		pRS415	CEN	LEU		This study
pJG1317	*HAP4*		*HAP4-LACZ*		pRS415	CEN	LEU		This study
pJG1318	*NDI1*		*NDI1-LACZ*		pRS415	CEN	LEU		This study
pJG1320	*QCR6*		*QCR6-LACZ*		pRS415	CEN	LEU		This study
pJG1321	*LAC1*		*LAC1-LACZ*		pRS415	CEN	LEU		This study
pJG1322	*LAG1*		*LAG1-LACZ*		pRS415	CEN	LEU		This study
pJG1335	*CBF1*		T211A-Cbf1 into pJG725		pRS416	CEN	URA		This study
pJG1336	*CBF1*		T212A-Cbf1 into pJG725		pRS416	CEN	URA		This study

Primer		Sequence			
JG3440		GGCCTCGAGCATTTTCTGTCGCTCTTATGATCC							
JG3441		GGCAAGCTTTGTCCACATAGCTCTTGTTTATTGA							
JG3442		GGCCTCGAGCCTCTTGTCCATTATCTTCGGGA							
JG3443		GGCAAGCTTTGATGTCATGTTGTCGTTATTTCTTC							
JG3457		GGCCTCGAGGTTGCTGTCATTCTTGATGACG							
JG3458		CGAATTCATGAAGGGGCAGCAGAAAACAGCTG							
JG3652		GGCCTCGAGGTATAATGTACCTTTGTTCCCTACAC							
JG3683		GGCAAGCTTATGACCATGATTACGGATTCACTGG							
JG3684		GGCGGATCCTTTTTGACACCAGACCAACTGGTAATG							
JG3669		GGCAAGCTTTAGAAAAGTCTTTGCGGTCATGATTC							
JG3670		GGCCTCGAGTTTCATCAAGGACTAAAGTTTTCTTATG							
JG3671		GGCAAGCTTTGATACAATTCTTGACAACATGACTAC							
JG3672		GGCCTCGAGGGCAAGCTTGACCTTTACTTTCCTCTCTAAAAGCC							
JG3673		GGCAAGCTTTTCCAACATGCCCATTTTCTATTTC							
JG3674		GGCCTCGAGCGAACAGCACAAGACGCGTATCAC							
JG3675		GGCAAGCTTTAGTGAAAGCATTGTGCTTGTTATC							
JG3676		GGCCTCGAGGCGGATTCCAAAGGCGTCCTTC							
JG3679		GGCAAGCTTCTTCGATAGCATAGTGGTTTTTAG							
JG3680		GGCCTCGAGGACCGGCGCTACCCGGTTAAG							
JG3741		GGCAAGCTT GATGTAGGGCATTACTTCTTTGATG							
JG3799		GGAAGAAATGTTTATCACCAGATGGAGAACCAATGAGCGG							
JG3800		CCGCTCATTGGTTCTCCATCTGGTGATAAACATTTCTTCC							
JG3801		GTCGTATTATTGAGTCACCAGCTTTATTCCATGGCGGG							
JG3802		CCCGCCATGGAATAAAGCTGGTGACTCAATAATACGAC							

### In vitro kinase assays

Full-length HIS-tagged yeast Psk1 (pJG858), Psk2 (pJG173) and Ugp1 (pJG210) proteins were purified from yeast (JGY4), while Cbf1 (pJG1031) and USF1 (pJG1233) proteins were from BL21DE3 *E. coli* (JHG504) as previously described (DeMille *et al.*, 2015) using Ni-NTA (Qiagen, Chatsworth, CA) chromotography. hPASK was expressed in Sf9 insect cells using the BAC-to-BAC baculovirus expression system (GIBCO/BRL) as previously described (Rutter *et al.* 2001) and purified using Ni-NTA (Qiagen, Chatsworth, CA) chromatography.

For yeast *in vitro* kinase assays, purified proteins were incubated with and without Psk1 in a 30 uL reaction containing 1x kinase buffer as previously described (DeMille *et al.*, 2014). For *in vitro* kinase assays using purified USF1 and hPASK proteins, reactions were run similar to the yeast proteins except for the following: 1 mM ATP was used and reactions were incubated for 30 min. Ipp1 (expressed from plasmid pJG1025) was purified similarly as Cbf1 and USF1, and was used as a negative control to show specificity of hPASK with USF1.

### Mitochondrial respiration

Yeast strains not transformed with a plasmid (wild type (JGY43), *psk1psk2* (JGY1244), *psk1* (JGY1348) and *psk2* (JGY1349)) were grown in YPAD overnight, diluted 1:100 in YPAGly/EtOH and grown for 13 hr. Wild type yeast (JGY43) transformed with an empty vector (pJG725), or *CBF1*-deficient yeast (JGY1227) transformed with either empty vector (pJG725), wild type Cbf1 (pJG1125), T211A-Cbf1 (pJG1335), T212A-Cbf1 (pJG1336), or USF1 (pJG1246) were grown in selective SD-Ura media until saturated, diluted 1:100 in SGly/EtOH-Ura and grown an additional 24-26 hr. The OD600 was taken to ensure equal growth among strains. In experiments requiring the *CBF1*-deficient yeast, yeast were freshly streaked from the freezer 48 hr prior to use to avoid selection of suppressors which were commonly seen. High-resolution O_2_ consumption was determined at 37° using the Oroboros O_2_K Oxygraph (Innsbruck, Austria). Samples were centrifuged at 5000 × g for 5 min and resuspended in warm mitochondrial respiration buffer 05 (MiR05; 0.5 mM EGTA, 10 mM KH2PO4, 3 mM MgCl2-6 H2O, 60 mM K-lactobionate, 20 mM HEPES, 110 mM sucrose, 1 mg/ml fatty acid free BSA, pH 7.1). After addition of sample, the chambers were hyperoxygenated to ∼350 nmol/ml. Following this, routine respiration was determined by measuring O_2_ consumption in the absence of any substrate (routine). Next, EtOH was added and then the uncoupler carbonyl cyanide p-(trifluoromethoxy)phenylhydrazone (FCCP;70 µM) as a measure of maximal electron transport system capacity (E). Finally, respiration was inhibited by the addition of the cytochrome c oxidase inhibitor, azide (20 mM) eliciting a state of residual oxygen consumption (ROX), which provided a control for all values. Samples were run in triplicate and averaged.

### TEM imaging

Wild type (JGY43), *cbf1* (JGY1227) and *psk1psk2* (JGY1244) yeast were grown overnight in YPAD then diluted into YPAraffinose and grown until OD600 ∼0.5. Cell size was measured using a Moxi Flow micro cytometer (ORFLO Technologies, Hailey, ID). Permanganate fixation protocol described by Perkins and McCaffery (Perkins and McCaffery 2007) was followed. Samples were sectioned at 80 nm using a RMC MTX ultramicrotome with a diamond knife then post stained with Reynold’s Lead Citrate for 10 min. Cells were observed in a Tecnai T-12 transmission electron microscope and images recorded digitally. Mitochondrial quantification was determined using AxioVision Rel 4.8 Software (Zeiss) as described by Braun *et al.* (Braun *et al.*, 2006). Each strain was examined in duplicate with wild type n = 74, *cbf1* n = 73, *psk1psk2* n = 69 images total per yeast strain obtained with the following criteria: 1. the image of the cell must be at least 3 um across to ensure the slice included a majority of the cell 2. the cell image must bear a visible nucleus 3. the cell image must appear to have an intact cell wall and 4. the cell image must be fairly uniform in shape to exclude cells that are budding.

### Mitochondrial isolation

Wild type (JGY 43), *cbf1* (JGY1227) and *psk1psk2* (JGY1244) yeast were grown in triplicate overnight in YPAD, diluted 1:100 into YPAGly/EtOH, and grown until OD600 ∼1.0-2.0. Preparation of Isolated Mitochondria by Differenting Centrifugation (Diekert *et al.*, 2001) was followed with the exception of Lyticase (Sigma) being used in place of Zymolase. Mitochondria were quantified using the Bradford assay. For mass spectrometry analysis, 10 uL of sample were diluted with 6X SDS sample buffer to a final concentration of 1X, and samples were separated on 12% SDS-PAGE until the loading dye had migrated approximately 1 cm. Bands were then obtained using a razor blade, and submitted to the UCSD Biomolecular and Proteomics Mass Spectrometry Facility for in-gel digestion and mass spectrometry analysis. One sample from JGY1227 failed to give data and was resubmitted for mass spectrometry analysis in solution (without being run on SDS-PAGE).

### In gel digest of MS samples

Gel slices were cut to 1 mm by 1 mm cubes and destained 3 times by first washing with 100 ul of 100 mM ammonium bicarbonate for 15 min, followed by addition of the same volume of acetonitrile (ACN) for 15 min (Shevchenko *et al.*, 1996). The supernatant was removed and samples were dried in a speedvac. Samples were then reduced by mixing with 200 µl of 100 mM ammonium bicarbonate-10 mM DTT and incubated at 56° for 30 min. The liquid was removed and 200 ul of 100 mM ammonium bicarbonate-55 mM iodoacetamide was added to gel pieces and incubated at room temperature in the dark for 20 min. After the removal of the supernatant and one wash with 100 mM ammonium bicarbonate for 15 min, the same volume of ACN was added to dehydrate the gel pieces. The solution was then removed and samples were dried in a speedvac. For digestion, enough solution of ice-cold trypsin (0.01 ug/ul) in 50 mM ammonium bicarbonate was added to cover the gel pieces and set on ice for 30 min. After complete rehydration, the excess trypsin solution was removed, replaced with fresh 50 mM ammonium bicarbonate, and left overnight at 37°. The peptides were extracted twice by the addition of 50 µl of 0.2% formic acid and 5% ACN and vortex mixing at room temperature for 30 min. The supernatant was removed and saved. A total of 50 µl of 50% ACN-0.2% formic acid was added to the sample, which was vortexed again at room temperature for 30 min. The supernatant was removed and combined with the supernatant from the first extraction. The combined extractions were analyzed directly by liquid chromatography (LC) in combination with tandem mass spectroscopy (MS/MS) using electrospray ionization.

### Preparation of MS samples in solution

Protein samples were diluted in TNE (50 mM Tris pH 8.0, 100 mM NaCl, 1 mM EDTA) buffer. RapiGest SF reagent (Waters Corp.) was added to the mix to a final concentration of 0.1% and samples were boiled for 5 min. TCEP (Tris (2-carboxyethyl) phosphine) was added to 1 mM (final concentration) and the samples were incubated at 37° for 30 min. Subsequently, the samples were carboxymethylated with 0.5 mg/ml of iodoacetamide for 30 min at 37° followed by neutralization with 2 mM TCEP (final concentration). Protein samples prepared as above were digested with trypsin (trypsin:protein ratio - 1:50) overnight at 37°. RapiGest was degraded and removed by treating the samples with 250 mM HCl at 37° for 1 h followed by centrifugation at 14000 rpm for 30 min at 4°. The soluble fraction was then added to a new tube and the peptides were extracted and desalted using C18 desalting columns (Thermo Scientific, PI-87782)(Guttman *et al.*, 2009; McCormack *et al.*, 1997; Shevchenko *et al.*, 1996).

### LC-MS/MS analysis

Trypsin-digested peptides were analyzed by ultra high pressure liquid chromatography (UPLC) coupled with tandem mass spectroscopy (LC-MS/MS) using nano-spray ionization. The nano-spray ionization experiments were performed using a TripleTof 5600 hybrid mass spectrometer (ABSCIEX) interfaced with nano-scale reversed-phase UPLC (Waters corporation nano ACQUITY) using a 20 cm-75 micrometer ID glass capillary packed with 2.5-µm C18 (130) CSH beads (Waters corporation). Peptides were eluted from the C18 column into the mass spectrometer using a linear gradient (5–80%) of ACN (Acetonitrile) at a flow rate of 250 μl/min for 1 h. The buffers used to create the ACN gradient were: Buffer A (98% H_2_O, 2% ACN, 0.1% formic acid, and 0.005% TFA) and Buffer B (100% ACN, 0.1% formic acid, and 0.005% TFA). MS/MS data were acquired in a data-dependent manner in which the MS1 data were acquired for 250 ms at m/z of 400 to 1250 Da and the MS/MS data were acquired from m/z of 50 to 2,000 Da. The Independent data acquisition (IDA) parameters were as follows; MS1-TOF acquisition time of 250 msec, followed by 50 MS2 events of 48 msec acquisition time for each event. The threshold to trigger MS2 event was set to 150 counts when the ion had the charge state +2, +3 and +4. The ion exclusion time was set to 4 sec. Finally, the collected data were analyzed using Protein Pilot 4.5 (ABSCIEX) for peptide identifications for in gel samples, or Protein Pilot 5.0 (ABSCIEX) for the single sample submitted in solution.

### Mitochondrial protein western blot

Mitochondria was isolated from wild type (JGY 43), *cbf1* (JGY1227) and *psk1psk2* (JGY1244) yeast using the same method listed previously. Protein concentration was determined using the Bradford protein assay. An equal amount of protein was loaded to each well of a 10% SDS-PAGE gel, separated, then transferred onto a nitrocellulose membrane. After incubation with 5% nonfat milk in TBST, the membrane was washed two times with TBS and probed with the selected antibody: Atp3 (the gamma subunit of the F_1_ sector of the F_0_F_1_ATP synthase, 1:5000, Invitrogen), Qcr7 (Subunit 7 of ubiquinol cytochrome-c reductase, complex III, 1:5000, a generous gift from Dr. Martin Ott, (Gruschke S., *et al.*, 2012)), Cox13 (Subunit VIa of cytochrome c oxidase, complex IV, 1:1000, a generous gift from Dr. Martin Ott), and porin (1:5000, Abcam). Membranes were washed two times with TBST and once with TBS then incubated with a 1:1000 dilution of horseradish peroxidase-conjugated anti-mouse or anti-rabbit antibodies for 2 hr. Blots were rinsed and then developed using the WesternBright ECL kit (Advansta Inc) according to the manufacturer’s protocol. Each blot was stripped by washing the blot in Amresco gentle-review stripping buffer for 30 min and probed a second time with a different antibody following the steps listed above. We ensured data were collected for each antibody on both a fresh blot and a stripped blot, except for Atp3 which was only done on a fresh blot due to sensitivity to the stripping buffer. Each biological replicate (n=≥4) was run in duplicate as a technical control. Bands were quantified using the ImageJ software.

### β-galactosidase reporter assays

A LacZ reporter plasmid (pJG1314) was constructed by amplifying LacZ from BL21DE3 *E. coli* with primers JG3683 and JG3684, digesting with *Hin*dIII/*Bam*HI and ligating into a similarly digested pRS415 vector (pJG121). Promoter regions (*LAC1* (JG3440/JG3441), *LAG1* (JG3442/JG3443), *ATP3* (JG3671/JG3672), *COX4* (JG3675/JG3676), *HAP4* (JG3669/JG3670), *NDI1* (JG3679/JG3680), and *QCR6* (JG3673/JG3674)) were amplified from wild type yeast template (JGY43) and cloned into the *Xho*I/*Hin*dIII sites of pJG1314. Yeast strains (wild type (JGY43), *cbf1* (JGY1227) *psk1psk2* (JGY1244) and *psk1psk2cbf1* (JGY1261)) were transformed with LacZ fusion plasmids containing each promoter region (pJG1321, (p*LAC1*-LacZ), pJG1322 (p*LAG1*-LacZ), pJG1315 (p*ATP3*-LacZ), pJG1316 (*pCOX4*-LacZ), pJG1317 (p*HAP4*-LacZ), pJG1318 (p*NDI1*-LacZ), pJG1320 (p*QCR6*-LacZ), grown in selective SD-Leu media for 2 days, diluted 1:50 in fresh media and grown until OD600 ∼0.5-1.0. β-galactosidase assays were performed as previously described by Stebbins and Triezenberg (Stebbins and Triezenberg 2004). Cell extracts were normalized by the Bradford assay to ensure equal amounts of total protein were used. Each strain was analyzed six times and averages were determined.

### Gel shift assays

Promoter regions for *LAC1(*JG3440/JG3441), *LAG1* (JG3442/JG3443), *ATP3* (JG3671/JG3672), and *PSK1* (JG3741/JG3652) were PCR amplified from wild type yeast template (JGY43). ATP3* and PSK1* promoter binding mutants were made by site-directed mutagenesis at the Cbf1 binding site using oligos JG3799/JG3800 (ATP3*) and JG3801/JG3802 (PSK1*) and were PCR amplified. Reaction mixes (20 ul) contained the PCR amplified promoter region, either 0 ng, 3 ng, or 6 ng of purified Cbf1 protein, and gel shift binding buffer (10 mM tris (pH 7.4), 1 mM EDTA, 50 mM KCl). Reaction mixes were incubated at room temperature for 20 min and analyzed by electrophoresis using 2% agarose gels.

### ATP assays

Wild type (JGY43), *cbf1* (JGY1227) and *psk1psk2* (JGY1244) yeast were grown overnight in YPAD at 30°, then shifted to room temperature for 24 hr (to avoid suppressor accumulation in the *cbf1* strain through prolonged growth at 30°) before dilution 1:50 into fresh YPAD and grown for 2 hr. Cultures were then centrifuged and resuspended in 5 mL YPA-Glycerol/EtOH and grown until OD600 ∼0.3-0.5, about 4 hr. ATP was then measured using the Promega Bac-titer Glo bioluminescent assay. A standard was generated using a known concentration of ATP provided in the kit. ATP levels were normalized to viable cell counts by plating diluted cells on YPAD.

### ROS assays

Flow cytometry was utilized to quantify the total intracellular reactive oxygen species (cell-ROS) and mitochondrial total reactive oxygen species (mit-ROS) adapted from (Perez *et al.*, 2014). The oxidant sensitive, cell permeant fluorescent probes H_2_DCFDA(Invitrogen) and dihydrohodamine 123 (DHR123, Invitrogen) were used to measure cell-ROS and mit-ROS respectively. Strains were grown in 2% YPAD overnight and then diluted 1:50 in YPAraffinose and grown for 4 hr. Samples were aliquoted and immediately, loaded with either H_2_DCFDA or DHR123 and incubated at 30° for 2 hr in the dark. Cells were then diluted with PBS buffer and immediately quantified by flow cytometry. The fluorescence was monitored in the emission fluorescence channel FL1. The populations of cells were gated in the forward scatter and side scatter dot plots to eliminate dead cells and cellular debris. Flowjo analytical software was used in order to quantify ROS levels between the strains.

### Respiration plate assays

Wild type yeast transformed with an empty vector (pJG725) or *cbf1* yeast (JGY1227) transformed with plasmids containing wild type Cbf1 (pJG1125), the empty vector, or USF1 (pJG1246) were grown 2 days in SD-Ura liquid media. Cultures were serially diluted (1/10) in water and spotted to both selective synthetic glycerol/EtOH, raffinose, or glucose plates (control) and incubated for 2-3 days at 30°.

### Data Availability Statement

The authors affirm that all data necessary for confirming the conclusions of this article are represented fully within the article, its tables and figures and the supplemental files. Supplemental material available at Figshare: https://doi.org/10.25387/g3.6933335.

## Results

### Psk1 inhibits respiration through phosphorylation of Cbf1 at T211

**Figure 1:**
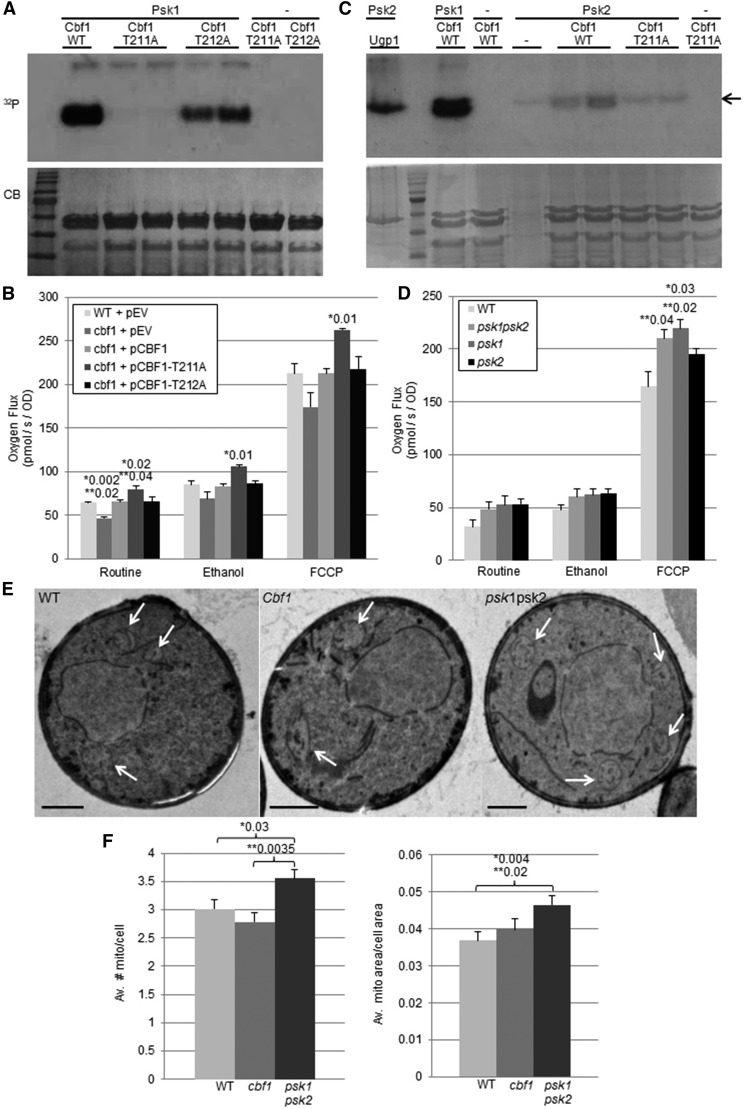
Psk1-dependent phosphorylation of Cbf1 at T211 controlled respiration. (A & C) *In vitro* kinase assays using purified Psk1 or Psk2, and Cbf1 wild type (WT) or mutant proteins incubated with radiolabeled-ATP. Ugp1 was a positive Psk2 control and Cbf1 purification yielded three bands visible in the Coomassie Brilliant Blue (CB) panel. Kinase reactions were separated by SDS PAGE, stained CB and imaged using autoradiography (^32^P). Black arrow in C indicates upper Psk2 autophosphorylation band. (B) WT or *CBF1*-deficient (*cbf1)* yeast transformed with indicated plasmids were grown in S-glycerol/EtOH media and respiration rates measured using an Oroboros O_2_K Oxygraph. Routine respiration was measured, then ethanol added, and finally, an uncoupler (FCCP) was added to represent maximal respiration. (D) WT, *psk1psk2*, *psk1* or *psk2* yeast were grown in YPAglycerol/EtOH and respiration measured as in B. (E) Representative pictures from transmission electron microscopy of WT, *cbf1* and PAS kinase-deficient (*psk1psk2)* yeast. Each yeast strain was grown in duplicate, fixed with permanganate (Perkins and McCaffery 2007), stained with Reynold’s Lead Citrate, then observed in a Technai T-12 transmission electron microscope. White arrows indicate mitochondria. Scale bars= 1uM. (F) Quantification of electron micrographs was determined using AxioVision Software (74 images were used for WT, 73 for *cbf1*, and 69 for *psk1psk2*). Error bars represent SEM. Significant p-values of data analyzed using pair wise Student’s T-Test(*) and one-way analysis of variance (ANOVA) with Tukey’s HSD *post hoc* test (**) are shown.

In our recent publication, the combination of threonines 211 and 212 of Cbf1 were important for Psk1 phosphorylation and inhibition of respiration (DeMille *et al.*, 2014). We further investigated each of these two sites individually (T211A and T212A) through *in vitro* kinase assays. Mutation of the T211 site dramatically reduced the ability of Psk1 to phosphorylate Cbf1, suggesting that it is the critical site for Psk1 phosphorylation (Figure 1A). Additionally, *CBF1*-deficient yeast transformed with the T211A mutant displayed a higher respiratory rate *in vivo* compared to both wild type *CBF1* and the T212A phosphomutant (Figure 1B). Both phosphomimetic forms of T211 (T211D and T211E) were not significantly different from the controls (data not shown), suggesting that the phosphomimetic mutations do not mimic Psk1 phosphorylation. The phosphomimetic form of UDP-glucose pyrophosphorylase (Ugp1), a well-characterized substrate of yeast PAS kinase, also did not mimic phospho-Ugp1 (Smith and Rutter 2007).

### Evidence for respiratory control as a specialized function of PAS kinase 1 (Psk1)

In budding yeast there are two orthologs of PAS kinase, Psk1 and Psk2 (Rutter *et al.*, 2001), which have high sequence similarity and are partially redundant in function (Rutter *et al.*, 2002). Many duplicated genes arose from a whole genome duplication event that occurred in an early ancestor of yeast (Byrne and Wolfe 2007; Conant and Wolfe 2007; Grassi *et al.*, 2010; Maclean and Greig 2011; Sugino and Innan 2005). From this, duplicated proteins either gained accessory functions allowing for their selection and maintenance, or were selected against and lost. The conservation of both Psk1 and Psk2 suggests differential functions for these proteins, however, both proteins phosphorylate the well-characterized substrate Ugp1 (Smith and Rutter 2007). To test whether Cbf1 is a substrate of both Psk1 and Psk2, *in vitro* kinase assays were performed (Figure 1C). Only weak phosphorylation of wild type Cbf1 by Psk2 was observed (lower band) suggesting that Psk1 is the major intracellular regulator of Cbf1. Note that Psk1 and Psk2 autophosphorylation was observed (upper ^32^P bands) and Psk2-dependent phosphorylation of Ugp1 (its known substrate) was observed, indicating that the Psk2 protein was active. No phosphorylation of Cbf1 was seen previously when using the kinase dead mutant of PAS kinase as control (DeMille *et al.* 2014). *In vitro* kinase assays, however, can be artifactual due to misfolded protein, the presence of protein contaminants, differential activities due to purification artifacts etc, making *in vivo* assays for Psk2 respiratory function a necessary verification (Figure 1D). We recently showed that *PSK1PSK2*-deficient yeast have an increased respiration rate, while *CBF1*-deficient yeast display a significant decrease when compared to wild type yeast (DeMille *et al.*, 2014). To determine whether this respiratory control is a specialized function of Psk1, we assayed respiration rates in the individual mutant yeast (*psk1* and *psk2*) compared to wild type and the double mutant (*psk1psk2*) (Figure 1D). Both *psk1psk2* and *psk1* yeast had significantly higher maximal respiration rates (respiration in the presence of the uncoupler FCCP) compared to wild type. There was no significant difference between *psk2* and wild type yeast. These *in vivo* results are consistent with the apparent decrease of Psk2 protein under respiratory conditions (Grose *et al.*, 2007) as well as the decreased *in vitro* phosphorylation of Cbf1 by Psk2 (Figure 1C), and support Psk1 as the primary ortholog responsible for regulating respiration in yeast.

### PAS kinase-deficient yeast have increased mitochondrial area

The observed effects on respiration could be due to several different alterations in the cell including changes in mitochondrial mass. To test the hypothesis that PAS kinase decreases respiration by down-regulating mitochondrial biogenesis pathways, mitochondria were viewed by transmission electron microscopy and both the number and total area of mitochondria were quantified in wild type, *cbf1* and *psk1psk2* yeast (Figure 1E & 1F). *PSK1PSK2*-deficient yeast showed a significant increase in both the number of mitochondria per cell as well as average mitochondrial area per total cell. These PAS kinase effects are consistent with the respiration phenotype; however, Cbf1 only trended toward a decreased number of mitochondria per cell without reaching significance. Thus, PAS kinase appeared to affect respiration by additional Cbf1-independent pathways in both the assays for mitochondrial area and the respiration assays (Figure 1F, and DeMille *et al.*, 2014).

### Mass spectrometry reveals a dramatically altered mitochondrial proteome in CBF1-deficient yeast

The fact that there was no obvious, statistical difference between mitochondrial number or area in the wild type and *CBF1*-deficient yeast suggests other mechanisms of respiratory regulation. In order to assess the major differences in the mitochondrial proteome of *CBF1*-deficient yeast, mitochondria were purified from wild type, *CBF1*-deficient, and *PSK1PSK2*-deficient yeast in triplicate, and subjected to analysis by mass spectrometry (see supplementary data 1 files for all total proteins retrieved). Surprisingly, although no difference in mitochondrial area was seen on a per cell basis when assessed by electron microscopy, the total amount of mitochondrial protein harvested per 0.5 liter yeast cultures was greatly reduced, consistent with the previously observed growth defects on respiratory carbon sources (DeMille *et al.*, 2014). In fact, normalizing to total cells using a flow cytometer appeared not to control for this difference because the *CBF1*-deficient yeast culture contains a large proportion of alive but very sick yeast. That is, when *CBF1*-deficient and wild type yeast are grown in glycerol/ETOH for six hours, normalized by OD600 and then plated on YPAD, at 48 hr there is huge decrease in *CBF1*-deficient colonies that arise. Thus, the ‘sick’ *CBF1*-deficient yeast may have more fragile mitochondria. This led to decreased detection of mitochondrial proteins in the *CBF1*-deficient samples when assessed by mass spectrometry, with the total number of proteins detected for wild type yeast (598+/−30.6 proteins) and *PSK1PSK2*-deficient yeast (580 +/− 62.4 proteins) being over seven times that from the *CBF1*-deficient yeast (79.7 +/− 11.5 proteins). Thus, a direct comparison of the total proteins retrieved from the three strains is hampered by the lower yield (the *CBF1*-deficient yeast lacked a majority of the mitochondrial proteins detected in the wild type and *PSK1PSK2*-deficient samples). To try to compensate for this discrepancy, we matched the number of proteins retrieved from the wild type and *PSK1PSK2*-deficient yeast directly to the *CBF1*-deficient yeast by comparing the same number of total proteins from the wild type and *PSK1PSK2*-deficient yeast (proteins were chosen starting with the highest confidence proteins until the same number of proteins were retrieved). This approach revealed several key differences between the strains. Although there are 51 proteins common to all three strains (42 of which are directly involved in respiration, Table S1), there are 50 additional proteins (26 directly involved in respiration) common to both the wild type and *PSK1PSK2*-deficient yeast that are not found in the *CBF1*-deficient yeast (this analysis is provided in Table S1).

**Figure 2:**
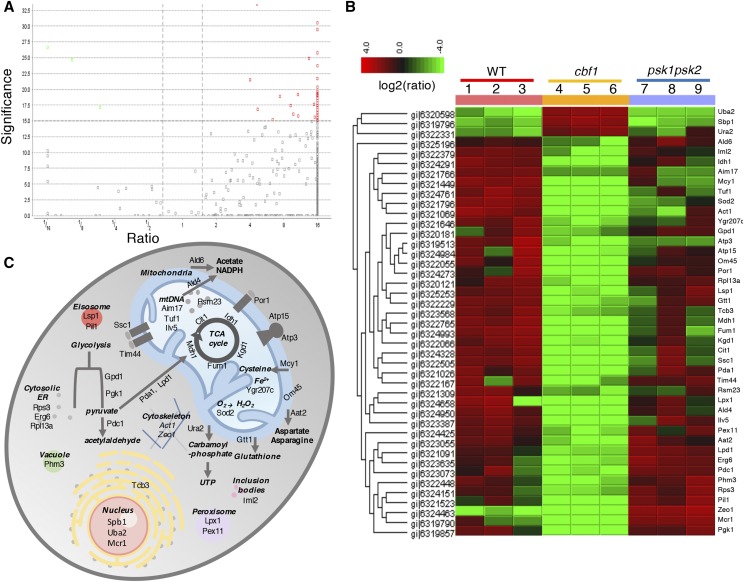
Comparison of the mitochondrial proteomes from WT, *cbf1* and *psk1psk2* yeast by mass spectrometry revealed 43 proteins with significantly altered expression. (A) Volcano plot identified 43 proteins whose peptides were significantly different when quantified by LC-MS/MS of purified mitochondria extracted from three independent samples of wild type (WT), *CBF1*-deficient (*cbf1*) and PAS kinase-deficient (*psk1psk2*) yeast grown in S-glycerol/EtOH media. Significance determined by ANOVA is provided on the y-axis, while log2(ratio) is given on the x-axis of the volcano plot. (B) A heat map of the 43 proteins with the protein sequence GI number provided on the left and the *S. cerevisiae* common protein name provided on the right. C) A figure representing the cellular location, and in some cases the function, of each of these 43 significantly altered proteins. Function and cellular localization were obtained from the *Saccharyomyces* Genome Database.

Volcano plots were also used to analyze the mass spectrometry data by plotting the ANOVA p-value and log2(ratio) of the fold difference of all proteins retrieved (Figure 2A). Forty-three proteins were identified as significantly different between the samples and their changes are presented as a heat map in Figure 2B. Of these 43 proteins, five were TCA cycle enzymes(Cit1, Idh1, *Kdg1*, Fum1 and Mdh1), two were ATP synthase Subunits (Atp3 and Atp15), several are mitochondrial transporters of small molecules (such as Ald4, Mcy1, Pda1, Lpd1), and two are involved in mitochondrial protein import (Tim44 and Ssc1). There were also several involved in the maintenance of mitochondrial DNA and reactive oxygen species (ROS) production and defense. The porin (Por1) protein, often used as an indication of total mitochondria, was also selectively reduced. In addition to these classic mitochondrial proteins, several glycolytic enzymes as well as enzymes known to associate with the outer mitochondrial membrane were found. A few enzymes associated with the peroxisome, cytosolic ER and nucleus were also reported. A figure representing the location, and in some cases the function, of each of these 43 hits is provided in Figure 2C. Of the 43 proteins, 40 appeared to have increased expression in the wild type *vs. CBF1*-deficient yeast (they are decreased in the knockout strain), while three appeared to have decreased expression.

**Figure 3:**
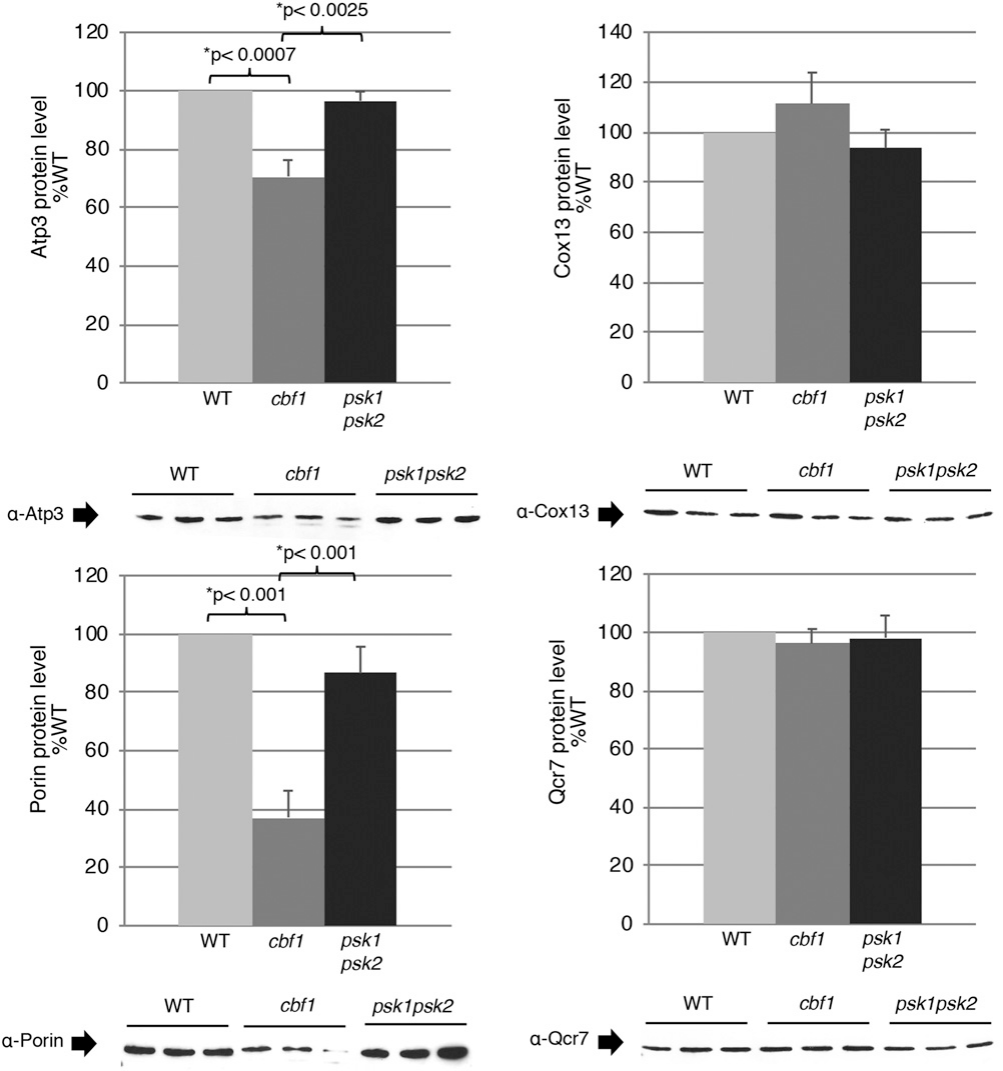
Quantification of Atp3, Qcr7, Cox13, and porin (Por1) protein in WT, *cbf1*, and *psk1psk2* isolated mitochondria. Each biological replicate (n=>4) was run in duplicate to control for technical issues. Representative western blots for each protein are shown below each graph. An equal amount of protein was loaded per sample as determined by Bradford assay with bovine serum albumin as a protein standard. Error bars represent SEM. Data were analyzed using the one-way analysis of variance (ANOVA) with Tukey’s HSD *post hoc* test. *P < 0.05.

Combined with the electron microscopy results, these results suggest that the respiration defect observed in *CBF1*-deficient yeast is in specific enzymes (such as the TCA cycle and ATP synthase subunits) rather than in an equal reduction of total mitochondrial proteins. To verify these findings, we performed western blot analysis of several common mitochondrial proteins and compared this to the levels of Atp3 (the gamma subunit of the F_1_ sector of the F_0_F_1_ATP synthase) and the porin protein, both predicted to have decreased expression from the mass spectrometry analysis. The western blot was normalized to total mitochondrial protein because mass spectrometry revealed many differences in proteins commonly used for normalization (such as porin). For each strain, the mitochondria was isolated (n>=4 independent isolations), total protein was quantified and the same amount of total protein was analyzed in technical duplicates to check loading. The Atp3 and porin protein levels appeared significantly reduced while electron transport proteins ubiquinol cytochrome-c reductase (Complex III) subunit 7 (Qcr7) and cytochrome c oxidase subunit VIa (Cox13) were not significantly altered (Figure 3). These results are consistent with the mass spectrometry analysis and suggest altered mitochondrial function rather than proportional reduction in mitochondrial area.

### Cbf1 regulates transcription of ATP3 and PSK1

**Figure 4:**
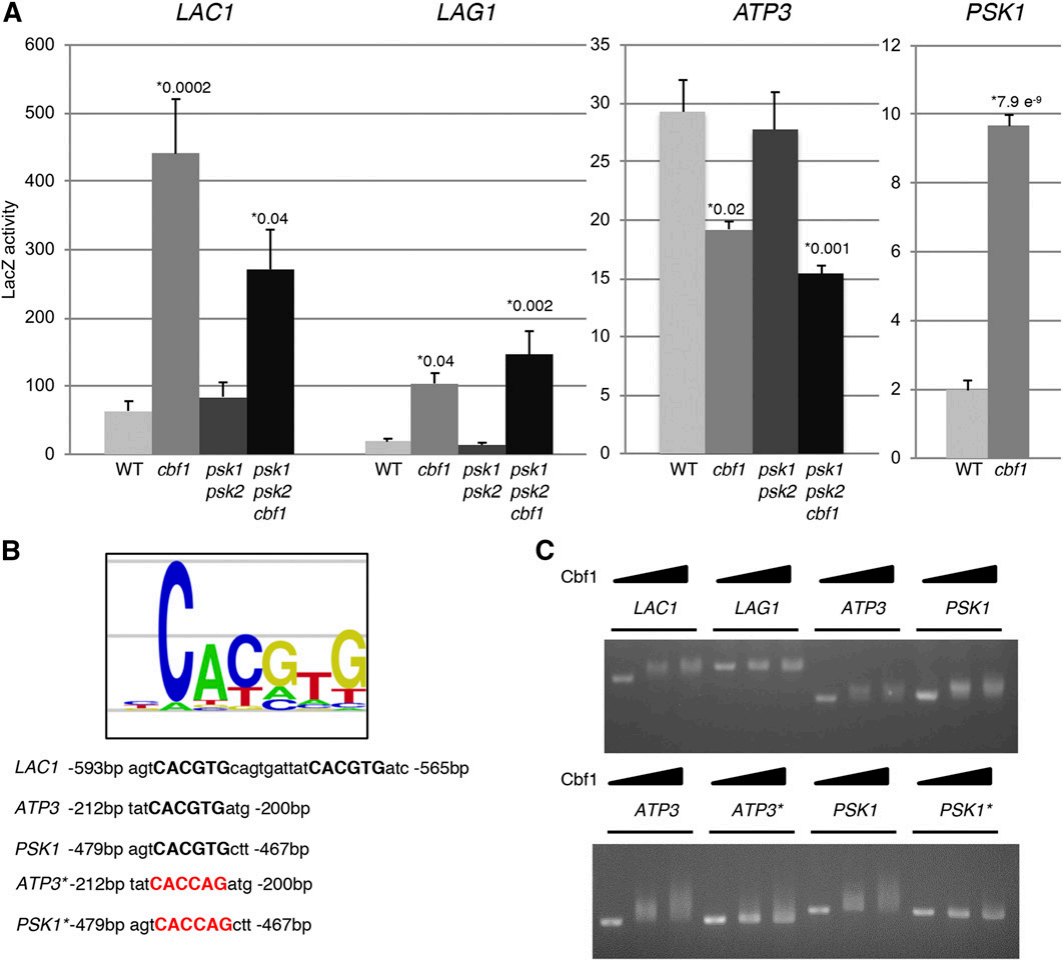
Evidence for the transcriptional regulation of *ATP3*, *LAC1*, *LAG1* and *PSK1* by Cbf1. (A) β-galactosidase assays of yeast transformed with LacZ fusion plasmids (to the *ATP3*, *LAC1*, *LAG1* or *PSK1* promoters) grown in synthetic minimal media. LacZ activity was used as a readout of transcriptional regulation. (B) A diagram of the Cbf1 consensus binding site found in *LAC1*, *ATP3* and *PSK1* upstream regions. *LAG1* does not harbor a Cbf1 binding site, therefore, the sequence is not shown. Mutant versions of the Cbf1 consensus in the *ATP3* and *PSK1* promoters were created by site-directed mutagenesis and are shown in red. (C) Gel shifts were carried out by PCR amplifying the promoter regions of *LAC1*, *LAG1*, *ATP3* and *PSK1* (top), as well as promoter regions with Cbf1 binding site alterations as controls (ATP3* and PSK1*) (bottom), then incubated with either 0 ng, 3 ng, or 6 ng of purified Cbf1 protein followed by agarose gel electrophoresis. Error bars represent SEM. Significant p-values for condition *vs.* WT are shown. Data were analyzed using one-way analysis of variance (ANOVA) and Tukey’s HSD *post hoc* test. Each strain was tested six times and averaged for β-galactosidase assays.

Large-scale transcription factor profiling (Lin *et al.*, 2013; MacIsaac *et al.*, 2006) has suggested Cbf1 as a potential player in respiratory control, potentially identifying the direct means by which Cbf1 controls respiration. We chose five of the respiration-associated genes reported as Cbf1-regulated in these large-scale transcriptome studies (*ATP3*, *COX4*, *HAP4*, *NDI1*, *QCR6)* and cloned their promoter regions into a LacZ fusion plasmid to test transcriptional control *in vivo*. Of the five genes tested, the gene encoding F_1_-ATP synthase complex *(ATP3*) was the only one that appeared to have increased expression due to Cbf1 under these growth conditions (Figure 4A). Notably, this was also the only protein of the five previously reported respiration genes that was also retrieved from the mass spectrometry proteome analysis discussed above. As a control for the assays, two ceramide synthase promoters (*LAC1* and *LAG1*) that were previously shown through β-galactosidase assays to be regulated by Cbf1 were also tested (Kolaczkowski *et al.*, 2004). The *LAC1* and *LAG1* behaved similarly to what Kolaczkowski *et al.* have reported, giving support that the assays were working properly. However, the lack of observing effects on the other four putative targets (*COX4*, *HAP4*, *NDI1*, or *QCR6*) may be due to unforeseen issues with the constructs, the yeast strain used, or growth condition differences. Taken together, these results are consistent with Cbf1 upregulating respiration (through transcriptional regulation of genes such as ATP3) and downregulating lipid biosynthesis (through regulation of genes such as *LAC1* and *LAG1*).

In addition to these five genes involved in respiration, *PSK1* was identified as a putative Cbf1 target from among the hundreds of putative targets identified in the large-scale transcription factor studies (Petti *et al.*, 2012). This suggested a feedback loop, where Psk1 phosphorylates and inhibits Cbf1, and Cbf1 in turn downregulates Psk1. The *PSK1* promoter was cloned into the LacZ fusion plasmid and also displayed Cbf1-dependent regulation (Figure 4A). These results support an important interplay between *CBF1* and *PSK1* and solidify the evolutionary link between them.

To test for direct binding of Cbf1 to the *ATP3* and *PSK1* promoters, gel shift assays were performed with purified promoters (DNA) and purified Cbf1 protein. As shown in Figure 4C, Cbf1 appears to bind the *ATP3*, *PSK1*, and *LAC1* promoters, but not the control (*LAG1*, which was previously shown to be regulated by Cbf1 in an indirect manner (Kolaczkowski *et al.*, 2004)). The predicted Cbf1 consensus site (CACGTG) was then mutated as shown in Figure 4B in order to confirm specificity of binding. Reduced binding was observed for both the mutated *ATP3* and *PSK1* promoters, supporting direct Cbf1 binding (Figure 4C).

### Altered ATP and ROS generation in CBF1-deficient cells

**Figure 5:**
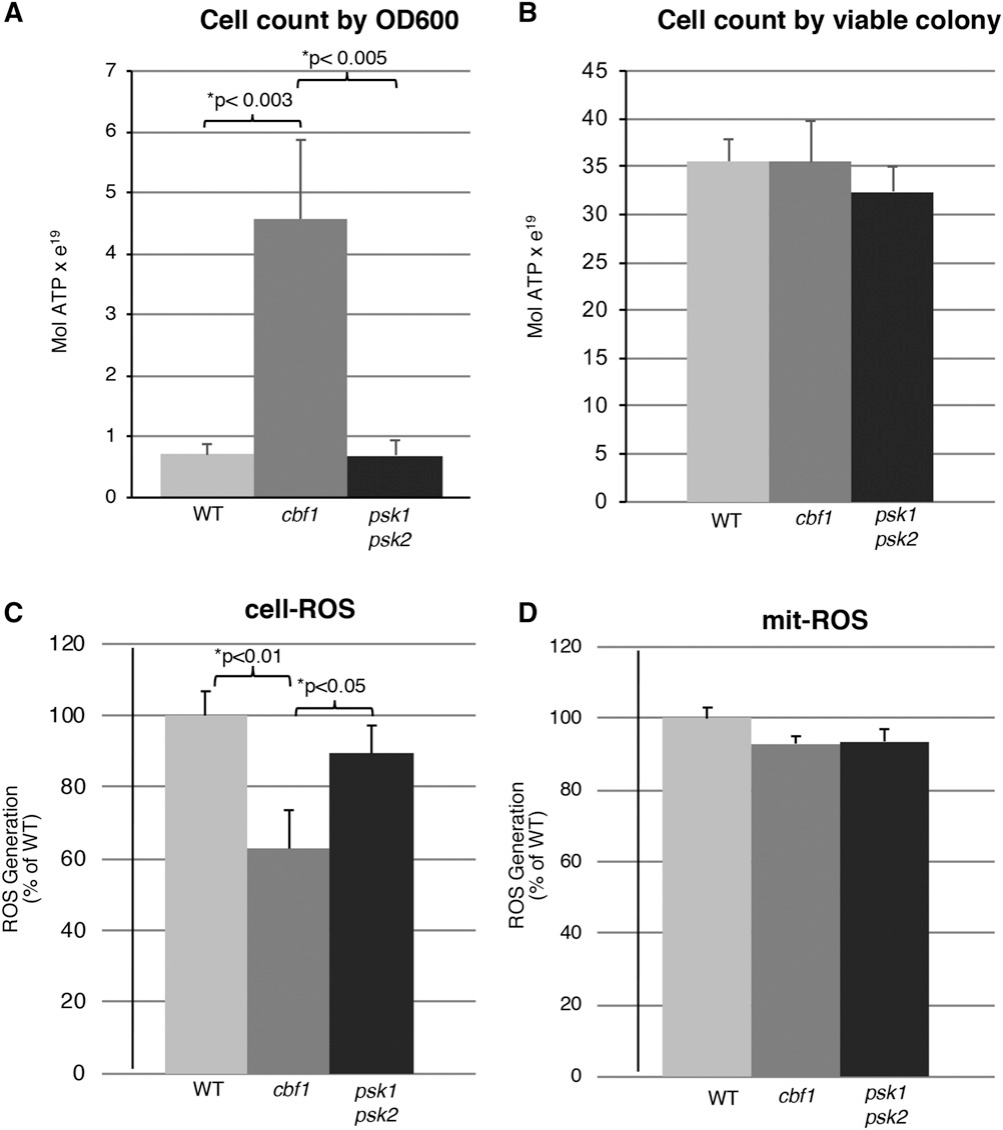
Cellular reactive oxygen species appeared to decrease in *CBF1*-deficient yeast while ATP assays revealed no significant change. ATP levels were measured in WT, *cbf1*, and *psk1psk2* yeast strains grown under respiratory conditions (Gly/EtOH) using Promega’s BactTiter-Glo Microbial Cell Viability Assay, a bioluminescent assay used for ATP quantification of bacterial or yeast cells. Yeast strains were normalized to either total cell count determined by OD600 (A) or by viable colonies from plating (B). Assays were run in technical duplicate and biological triplicate and results were averaged. Flow cytometry was utilized to quantify the (C) total intracellular reactive oxygen species (cell-ROS) and (D) mitochondrial reactive oxygen species (mit-ROS) in WT, *cbf1*, and *psk1psk2* yeast strains. The fluorescence was monitored in the emission fluorescence channel FL1. Error bars represent SEM. Significant p-values for condition *vs.* WT are shown with asterisks. *p-value was determined using one-way analysis of variance (ANOVA) and Tukey’s HSD *post hoc* test.

The transcriptional regulation results, in conjunction with the mass spectrometry findings, strongly suggest that Cbf1 upregulates *ATP3* to promote respiration. Therefore, we monitored ATP production in wild type, *CBF1*-deficient and *PSK1PSK2*-deficient yeast. Unexpectedly, the *CBF1*-deficient yeast displayed a drastically higher ATP level per cell when compared to the wild type or *PSK1PSK2*-deficient yeast when using OD600 to normalize the cell count (Figure 5A). This effect, however, appeared to be due to a large proportion of dead/unhealthy cells as seen with the mass spectrometry mitochondrial preparation above because there was no significant alteration in ATP when we normalized to total viable cell count determined by plating (Figure 5B). Next we investigated whether the altered respiratory metabolism of *CBF1*-deficient yeast resulted in reactive oxygen species (ROS) generation (Figure 5C & D). The *CBF1*-deficient strain appeared to have a lower level of ROS generation than wild type yeast, consistent with decreased respiration in these yeast and supporting the altered mitochondrial proteome discussed above.

### Human USF1 protein is a conserved substrate of PAS kinase in vitro and affects respiration in vivo

**Figure 6:**
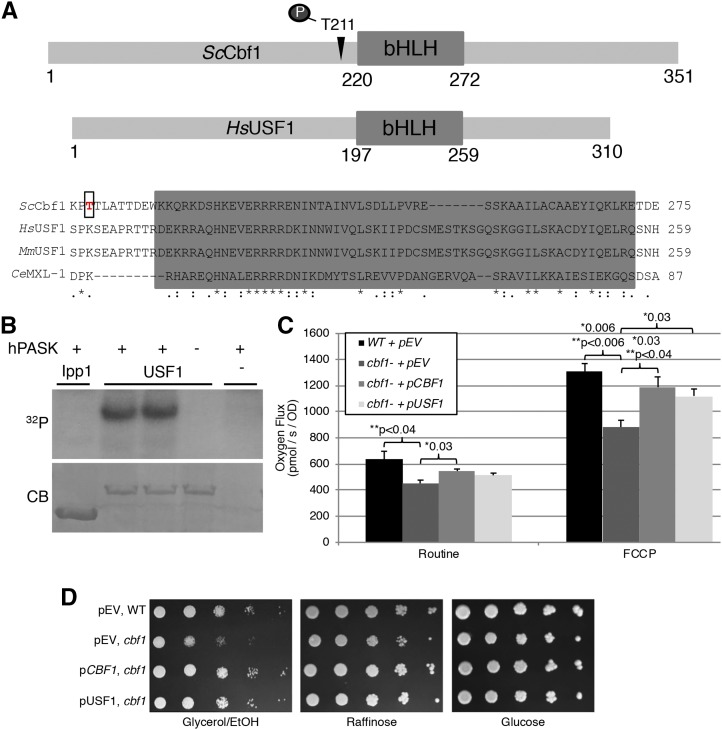
Evidence for the phosphorylation of human USF1 by hPASK and for a conserved role in the regulation of respiration. (A) Clustal Omega (Goujon *et al.*, 2010; Sievers *et al.*, 2011) alignment of the bHLH domain region for functional orthologs of Cbf1 obtained from the Isobase database (Liao *et al.*, 2009; Park *et al.*, 2011; Singh *et al.*, 2008): *S. cerevisiae* (*Sc*Cbf1), *H. sapiens* (*Hs*USF1), *M. musculus* (*Mm*USF1), and *C. elegans* (*Ce*MXL-1). (B) Kinase assays using purified hPASK and USF1 proteins were run on 12% SDS page, stained with Coomassie Brilliant Blue (CB), then imaged using autoradiography (^32^P). Ipp1, retrieved from a previous screen for PAS kinase interactors (DeMille *et al.*, 2014), was used as a negative control. (C) For respiration assays, overnights in selective S-glucose media were switched to selective S-galactose media and respiration rates were measured using an Oroboros O_2_K Oxygraph. (D) Wild type and *cbf1* yeast transformed with an empty vector, *CBF1* or USF1 were grown in selective S-glucose media. Overnights in selective S-glucose media were serially diluted in water (1:10) then plated to selective S-glycerol/EtOH, -raffinose, or -glucose plates and incubated for 2-3 days at 30°C. Significant p-values for data were analyzed using pair wise Student’s T-Test (*) and one-way analysis of variance (ANOVA) with Tukey’s HSD *post hoc* test (**) are shown.

Just as Cbf1 regulates lipids in yeast through transcriptional control of *LAC1* and *LAG1*, it’s human homolog Upstream transcription factor 1 (USF1) is a major contributor to familial combined hyperlipidemia (FCHL) (Auer *et al.*, 2012; Coon *et al.*, 2005; Di Taranto *et al.*, 2015; Holzapfel *et al.*, 2008; Huertas-Vazquez *et al.*, 2005; Komulainen *et al.*, 2006; Laurila *et al.*, 2016; Lee *et al.*, 2007; Meex *et al.*, 2008; Naukkarinen *et al.*, 2006; Naukkarinen *et al.*, 2005; Naukkarinen *et al.*, 2009; Ng *et al.*, 2005; Pajukanta *et al.*, 2004; Plaisier *et al.*, 2009; Reiner *et al.*, 2007; van der Vleuten *et al.*, 2007). These studies have provided strong evidence for the role of USF1 in lipid regulation, but a role in respiratory regulation has not been shown. Cbf1 and USF1 have homology in the region of the PAS kinase phosphorylation site on Cbf1, just upstream of the conserved basic helix-loop-helix domain (Figure 6A). To determine if USF1 is a conserved substrate of human PAS kinase (hPASK) *in vitro* kinase assays were performed (Figure 6B). USF1 was phosphorylated *in vitro* in a hPASK-dependent manner. The effects of USF1 on respiration were then assessed in yeast through both respiration chamber assays (Figure 6C) as well as plate assays (Figure 6D). The results gave supporting evidence that USF1 can complement the respiration phenotype of *CBF1*-deficiency in yeast, suggesting that Cbf1/USF1 play a conserved role in the partitioning of glucose toward respiration and away from lipid biogenesis.

## Discussion

**Figure 7:**
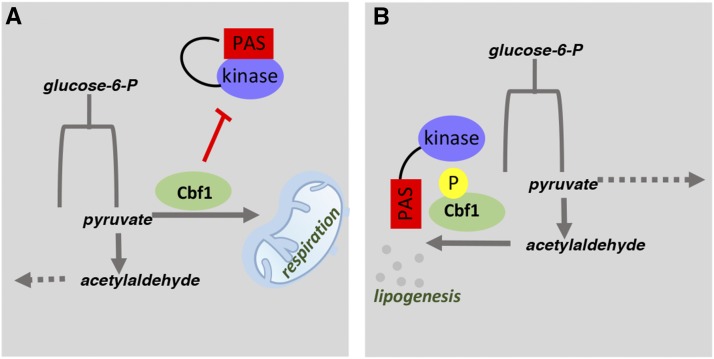
A model for function of Cbf1 and PAS kinase as a key point in the partitioning of glucose for respiration or for lipogenesis. A) When PAS kinase is inactive, Cbf1 upregulates pathways involved in respiration and inhibits PAS kinase expression. B) When PAS kinase is active, Cbf1 is phosphorylated at T211 and upregulates pathways involved in lipogenesis.

As the primary source of cellular energy, the regulation of mitochondrial metabolism is key in proper glucose allocation. As such, mitochondrial dysfunction has come to the forefront of a wide variety of disease including diabetes, obesity, Alzheimer’s and cancer (Cardoso *et al.*, 2009; Chow *et al.*, 2017; Hu *et al.*, 2017). Despite their clear importance, there is still much unknown about mitochondrial regulation. This study provides novel molecular mechanisms behind the regulation of mitochondrial metabolism by PAS kinase and its substrate Cbf1/USF1. Our results suggest that PAS kinase and Cbf1 function at a pivotal point for partitioning glucose to respiration or to lipid biosynthesis (Figure 7).

First, PAS kinase-dependent phosphorylation of Cbf1 at the critical threonine 211 site appears to reduce cellular respiration (Figure 1A-D). The mechanisms behind this respiratory phenotype were characterized by looking at mitochondrial area and the mitochondrial proteome. No significant decrease of mitochondrial area was observed by transmission electron microscopy of *CBF1*-deficient yeast (Figure 1E & F), suggesting alternative methods for regulating respiration. In contrast, PAS kinase-deficient yeast did display an increased mitochondrial area, suggesting additional targets that may control mitochondrial biogenesis as supported by the additional affects observed in the respiration assays (Figure 1 and DeMille *et al.*, 2014). This approximately one-third increase in area may help account for the increase in respiration, however it is difficult to know how much the mitochondrial area can directly correlate to function since the effects on mitochondrial proteins reported in this paper show that they may not be evenly regulated.

Although the effects of Cbf1 on respiration did not appear to be on mitochondrial area, an apparent significant effect on the mitochondrial proteome was detected by mass spectrometry, with 43 proteins that appeared significantly altered in *CBF1*-deficient yeast (Figure 2). These results indicated altered levels of key electron transport chain proteins, including a decrease in Atp3 (F_1_ ATP synthase) and porin levels which were confirmed by western blot (Figure 3).

Due to the presence of a conserved Cbf1-binding site in the ATP3 promoter, promoter β-galactosidase and gel shift assays were performed in parallel with genes (*LAC1*, *LAG1*) involved in lipid biosynthesis previously shown to be Cbf1-regulated (Kolaczkowski *et al.*, 2004; Petti *et al.*, 2012). Atp3 appeared to have *CBF1*-dependent increased expression and Cbf1 bound the *ATP3* promoter in the *in vitro* gel shift assays (Figure 4). Thus, Cbf1 appears to upregulate respiration through *ATP3* (Figure 2-4), and to downregulate lipid biogenesis through *LAC1* and *LAG1* (Figure 4).

The *CBF1*-dependent increase of *ATP3* expression suggested that ATP levels may be lower in *CBF1*-deficient yeast. However, we found that while *CBF1*-deficient yeast had lower ROS generation consistent with decreased respiration, no significant difference in ATP levels were observed when levels were normalized to cell count (Figure 5). These results suggest that *CBF1*-deficient yeast may produce a majority of the ATP in alternative pathways. Since 40 of the 43 high confidence hits from mass spectrometry displayed decreased expression in *CBF1*-deficient yeast, ATP could be produced by pathways not localized to the mitochondria (such as increased glycolysis).

In addition to Atp3, we confirmed PAS kinase 1 (*PSK1*) as a direct transcriptional target of Cbf1, solidifying the importance of their interaction (Figure 4). From promoter β-galactosidase assays, *CBF1*-deficiency led to an almost fivefold increase in Psk1 expression, and Psk1 in turn inhibits Cbf1 activity through phosphorylation (DeMille *et al.*, 2014). In addition, the activation of PAS kinase by respiratory growth conditions, and its subsequent inhibition of respiration appears contradictory. In each of these cases, PAS kinase and/or Cbf1 appears to be providing a feedback mechanism for delicately controlling metabolism, partitioning glucose to competing pathways and ensuring, for example, that some lipids are produced from glucose breakdown even during times when respiration is favored. This interplay between the two indicates a long evolutionary history, suggesting the pathway may be conserved in higher eukaryotes.

The human homolog of Cbf1, USF1, has been associated with lipid biogenesis and hyperlipidemia in many studies (Auer *et al.*, 2012; Coon *et al.*, 2005; Di Taranto *et al.*, 2015; Holzapfel *et al.*, 2008; Huertas-Vazquez *et al.*, 2005; Komulainen *et al.*, 2006; Laurila *et al.*, 2016; Lee *et al.*, 2007; Meex *et al.*, 2008; Naukkarinen *et al.*, 2006; Naukkarinen *et al.*, 2005; Naukkarinen *et al.*, 2009; Ng *et al.*, 2005; Pajukanta *et al.*, 2004; Plaisier *et al.*, 2009; Reiner *et al.*, 2007; van der Vleuten *et al.*, 2007). Combined with our results herein Cbf1/USF1 may play a conserved role in partitioning glucose toward respiration at the expense of lipid metabolism. In support of this hypothesis, USF1 appears to regulate respiration in yeast (Figure 6C & D). The role of PAS kinase in inhibiting Cbf1 also appears to be conserved, in that hPASK can phosphorylate USF1 *in vitro* (Figure 6B). The conservation of the USF1/PAS kinase pathway may aid in explaining the most dramatic phenotypes reported for PAS kinase-deficient mice, namely an increased O_2_ uptake and decreased liver triglyceride accumulation (Hao *et al.*, 2007). Further study of how PASK is regulating the many activities of USF1 may provide additional insight into the role of PAS kinase in human diseases including hyperlipidemia, obesity and diabetes.
